# Quadriceps muscle atrophy after non-invasive anterior cruciate ligament injury: evidence linking to autophagy and mitophagy

**DOI:** 10.3389/fphys.2024.1341723

**Published:** 2024-03-01

**Authors:** Sung Gi Noh, Ahram Ahn, Steven M. Davi, Lindsey K. Lepley, Oh Sung Kwon

**Affiliations:** ^1^ Department of Kinesiology, University of Connecticut, Storrs, CT, United States; ^2^ Cooperative Studies Program Coordinating Center (CSPCC), VA Connecticut Healthcare System, West Haven, CT, United States; ^3^ School of Kinesiology, University of Michigan, Ann Arbor, MI, United States; ^4^ Department of Orthopaedic Surgery and Center on Aging, University of Connecticut School of Medicine, Farmington, CT, United States

**Keywords:** ACL injury, autophagy, mitophagy, muscle atrophy, vastus lateralis muscle

## Abstract

**Introduction:** Anterior cruciate ligament (ACL) injury is frequently accompanied by quadriceps muscle atrophy, a process closely linked to mitochondrial health and mitochondria-specific autophagy. However, the temporal progression of key quadricep atrophy-mediating events following ACL injury remains poorly understood. To advance our understanding, we conducted a longitudinal study to elucidate key parameters in quadriceps autophagy and mitophagy.

**Methods:** Long-Evans rats were euthanized at 7, 14, 28, and 56 days after non-invasive ACL injury that was induced via tibial compression overload; controls were not injured. Vastus lateralis muscle was extracted, and subsequent immunoblotting analysis was conducted using primary antibodies targeting key proteins involved in autophagy and mitophagy cellular processes.

**Results:** Our findings demonstrated dynamic changes in autophagy and mitophagy markers in the quadriceps muscle during the recovery period after ACL injury. The early response to the injury was characterized by the induction of autophagy at 14 days (Beclin1), indicating an initial cellular response to the injury. Subsequently, at 14 days we observed increase in the elongation of autophagosomes (Atg4B), suggesting a potential remodeling process. The autophagosome flux was also augmented between 14- and 28 days (LC3-II/LC3-I ratio and p62). Notably, at 56 days, markers associated with the elimination of damaged mitochondria were elevated (PINK1, Parkin, and VDAC1), indicating a possible ongoing cellular repair and restoration process.

**Conclusion:** These data highlight the complexity of muscle recovery after ACL injury and underscore the overlooked but crucial role of autophagy and mitophagy in promoting the recovery process.

## 1 Introduction

Despite lengthy rehabilitation programs, many patients with anterior cruciate ligament (ACL) injury develop persistent atrophy and weakness of their quadriceps hallmarked by reductions in quadriceps volume, cross-sectional area, and muscle strength (Kobayashi et al., 2004; Stockmar et al., 2006; Marcon et al., 2015; Thomas et al., 2016; Cristiani et al., 2019; Lepley et al., 2019). The mechanisms underlying these changes are complex and involve a cascade of neurological and molecular events. So far, the majority of research has examined neurogenic-mediated pathways of quadriceps muscle weakness (Lepley and Lepley, 2022). Much less is known about the cellular and molecular aspects of muscle atrophy.

At the cellular level, ACL-injured humans have been shown to have reduced satellite cell content per muscle fiber, expansion of extracellular matrix owing to the accumulation of fibroblasts, and changes in muscle fiber type and size (Noehren et al., 2016; Fry et al., 2017). Blunted protein anabolism and a heightened inflammatory response have also been identified as critical factors mediating quadriceps muscle atrophy after ACL injury (Hunt et al., 2020; Keeble et al., 2023). These post-traumatic alterations suppress the growth of muscle, limiting the return to play of many patients and contributing to the development of early osteoarthritis (Oiestad et al., 2015). Though protracted quadriceps atrophy is a common outcome that afflicts many ACL patients, the gap between research and clinical solutions remains, as longitudinal work that details the natural history of disease progression is lacking.

Mitochondria are a promising therapeutic target due to their involvement in many key cellular functions that regulate muscle health (e.g., energy production, regulation of calcium levels, and reactive oxygen species [ROS] generation) (Russell et al., 2014; Mishra and Chan, 2016). Their health is largely dependent on the contributions of autophagy (i.e., conserved degradation of the cell that removes unnecessary or dysfunctional), mitophagy (i.e., autophagy that targets damaged or dysfunctional mitochondria) and mitochondrial biogenesis (i.e., synthesis of new mitochondria) (Palikaras and Tavernarakis, 2014; Ma et al., 2020), and when this dynamic is disrupted, muscle atrophy can occur. To this point, a growing body of evidence suggests that inhibiting autophagy leads to diminished mitochondrial oxygen consumption, increased ROS production, and poor adenosine triphosphate (ATP) synthesis capacity (Wu et al., 2009; Parousis et al., 2018; Bakula and Scheibye-Knudsen, 2020). The accumulation of defective mitochondria is also known to trigger a decrease in myofiber size, sarcoplasmic reticulum expansion, and abnormal membrane structure (Masiero et al., 2009). It is clear that autophagy and mitophagy play critical roles in the maintenance of muscle health, yet their contribution to the regulation of quadriceps muscle health after ACL injury is unknown.

The concept that mitochondrial dysfunction initiates or is involved in the detrimental sequela of muscle dysfunction is novel to ACL injury, but is well-established and consistent with other areas (Chen and Chan, 2009; Drew et al., 2014; Tang et al., 2014; Carter et al., 2018; van der Ende et al., 2018; Penna et al., 2019; Triolo and Hood, 2019; Memme et al., 2022). Indeed, our new breakthrough data show that muscle mitochondria may play a key role in regulating quadriceps muscle health after ACL injury, as we show long-lasting impairments in respiration capacity (i.e., respiratory control ratio [RCR], state 3, and state 4 respiration) and excessive levels of ROS production (i.e., by 30%–100%) post-injury (Davi et al., 2022). We also show that these impairments in maximal oxygen consumption (at state 3) are tightly coupled with a shift towards smaller and faster quadricep muscle fiber-types (Davi et al., 2022). Notably, these depressions in quadricep mitochondrial function occurred alongside dramatic alterations in knee flexion profiles that were directly associated with hallmark characteristics of knee osteoarthritis onset, demonstrating the functional significance of this sequelae (White et al., 2022). Importantly, early human data also show disrupted mitochondrial respiratory capacity with prolonged oxidative damage after ACL injury (Latham et al., 2024), making the important link between preclinical experiments and clinical outcomes. Though promising, in order to develop target-based therapies, it is imperative to more fully understand and define the role of muscle mitochondria after ACL injury. Thus, the purpose of this study was to elucidate the temporal progression of key quadricep muscle autophagy and mitophagy events after non-invasive ACL injury using our non-invasive preclinical model.

## 2 Materials and methods

### 2.1 Experimental animals and non-invasive ACL injury

This study protocols were approved by the University of Connecticut Institutional Animal Care and Use Committee (IACUC approval #A17-042) and followed the National Institutes of Health guidelines for the care and treatment of experimental animals through all procedures. Forty adult Long Evans rats (16 weeks of age, 20 males and 20 females) were purchased from Envigo Laboratories and housed in standard conditions on a 12 h:12 h light-dark cycle and temperature at 23°C ± 2°C with access to food and water *ad libitum*.

Our previous work within the same cohort has detailed the time course of biomechanical adaptations after ACL injury, establishing negative changes in knee movement quality (White et al., 2022). In the same cohort, we have also shown that there are long-lasting changes in mitochondrial respiratory function (Davi et al., 2022). As an extension of this work that shows clear functional abnormalities (Davi et al., 2022; White et al., 2022), we sought to explore the molecular underpinnings quadricep muscle autophagy and mitophagy events after injury. Accordingly, ACL injury was delivered to the right limb with a single load tibial compression to induce non-invasive ACL rupture that has been described elsewhere (White et al., 2022). Thirty-two rats underwent the non-invasive ACL rupture procedure, which was confirmed by trained investigators using Lachman’s test and post-mortem via dissection. All injured rats were euthanized at days 7, 14, 28, and 56 after ACL injury (4 males and 4 females per time point) and the right vastus lateralis of the quadriceps was collected and stored at −80°C for immunoblotting. Additionally, eight healthy rats (∼24 weeks of age, 4 males and 4 females) served as controls.

### 2.2 Immunoblotting

Frozen muscle samples were homogenized with a use of radioimmunoprecipitation assay (RIPA) lysis buffer (Santa Cruz Biotechnology, sc-24948A) containing a protease and phosphatase inhibitor (Sigma-Aldrich, PPC1010). The Bradford assay reagent (Thermo Fisher Scientific, 1863028) was utilized to quantify protein concentration. Briefly, equal amounts of lysate (22.5 μg) were loaded into polyacrylamide gels (Bio-Rad, 5678094) for electrophoresis and subsequently transferred onto polyvinylidene difluoride (PVDF) membrane (Bio-Rad, 10026933). Membranes were then blocked under 5% non-fat dry milk dissolved in Tris-buffered saline (pH 7.5) containing 0.1% Tween-20 (TBST; 10 x TBS [Bio-Rad, 1706435], 10% Tween-20 [Bio-Rad, 1610781]) for 1 h at room temperature, and incubated with primary antibodies overnight at 4°C. Secondary antibodies were adding the following day, and incubated on the membrane for 1 h at room temperature. The membranes were subsequently imaged with a ChemiDoc (Bio-Rad, 12003153) and protein quantification was performed with Image Lab software (Bio-Rad). The immunoblot images taken from membranes and used in Figures include a mixture of both male and female samples.

The primary antibodies were directed against proteins that play critical roles in autophagy and mitophagy. The proteins measured as markers for autophagy and mitophagy included the following: 1) Beclin1 (1:1000, Cell Signaling Technology, 3495S; Danvers, MA, USA) as an autophagy upstream marker, 2) autophagy-related (Atg)12 (1:1000, Cell Signaling Technology, 4180S), Atg4A (1:1000, Cell Signaling Technology, 7613S), Atg4B (1:1000, Cell Signaling Technology, 5299S), and Atg7 (1:1000, Cell Signaling Technology, 8558S) as pre-lysosomal markers, 3) microtubule-associated protein 1A/1B-light chain 3 (1:1000, LC3A/B; Cell Signaling Technology, 4108S) and p62 (1:1000, sequestosome 1; Sigma-Aldrich, P0067; St Louis, MO, USA) as autophagosome markers, 4) PTEN-induced putative kinase protein 1 (1:1000, PINK1; Cell Signaling Technology, 6946S), Parkin (1:1000, Cell Signaling Technology, 4211S), dynamin-related protein 1 (1:1000, Drp1; Abcam, Ab56788; Cambridge, MA, USA), and voltage-dependent anion channel 1/Porin (1:1000, VDAC1; Abcam, Ab14734) as markers for PINK1-Parkin-mediated mitophagy, and 5) lysosomal associated membrane protein (Lamp) 1 (1:1000, Abcam, Ab24170) and Lamp2 (1:1000, Abcam, Ab13524) as lysosomal markers. Glyceraldehyde-3-phosphate dehydrogenase (GAPDH; 1:5000, Cell Signaling Technology, 3683S) was utilized for protein normalization.

### 2.3 Statistical analysis

To elucidate the temporal progression of key quadricep muscle autophagy and mitophagy events after non-invasive ACL injury, two-way analyses of variance were performed with factors of time since ACL injury and sex (male/female) entered into the model. If there was no interaction effect, the main effect of time since injury and/or sex were evaluated. In the case of significant interactions, Least Significance Differences tests were used. Alpha level was set at *p* < 0.05 *a priori*. All data are expressed as mean ± SEM and were analyzed with SPSS software version 29.0 (IBM, Armonk, NY, USA).

## 3 Results

### 3.1 Autophagy upstream marker Beclin1 was elevated at 7 and 14 days and decreased afterwards

**FIGURE 1:**
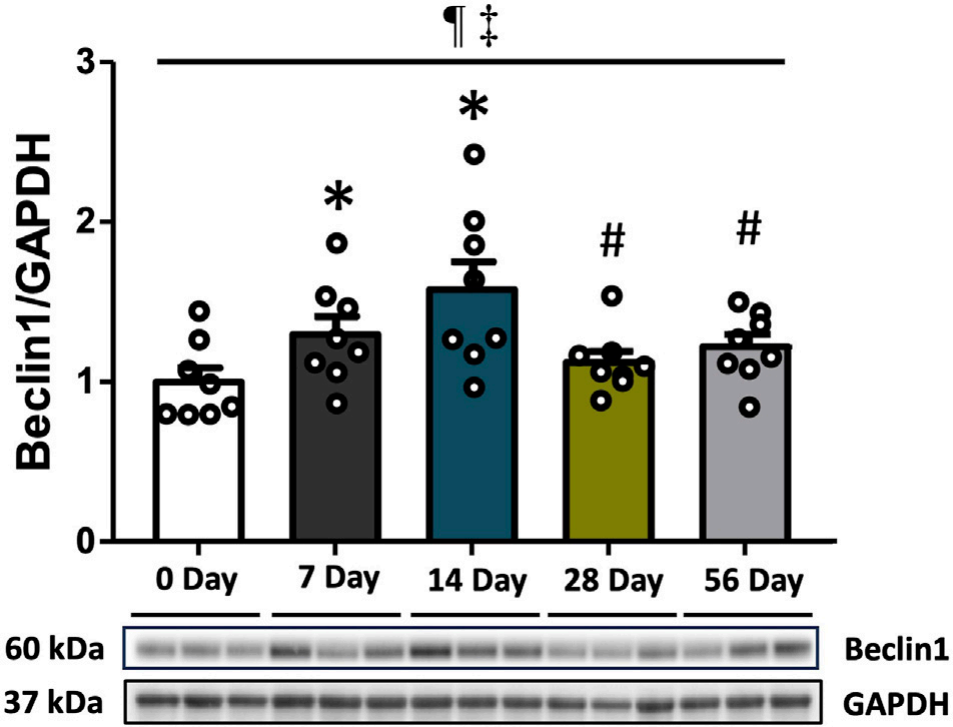
The upstream marker of autophagy following ACL injury. Beclin1 protein concentrations via Western blot analysis with supportive representative images. Beclin1 levels exhibited an increase at 7- and 14 days compared to CON, followed by a decrease at 28- and 56 days. Data are presented as Mean ± SEM. * Significant difference (*p* < 0.05) *versus* CON. # Significant difference (*p* < 0.05) *versus* 14 days. ¶ Significant main effect of time regardless of sex (*p* < 0.05). ‡ Significant main effect of sex regardless of time (*p* < 0.05).

Beclin1 protein expression was analyzed to investigate the induction of autophagy. Compared to the control group, Beclin1 levels showed a 1.3-fold increase at 7 days (*p* < 0.05) and a 1.6-fold increase at 14 days (*p* < 0.001). However, there was a subsequent decrease in Beclin1 levels at 28- and 56 days (*p* < 0.05, Figure 1). Regardless of time and injury status, males exhibited higher Beclin1 levels than females (*p* = 0.003, Supplementary Figure S1).

### 3.2 Pre-lysosomal marker Atg4B was augmented at 14 and 28 days

**FIGURE 2:**
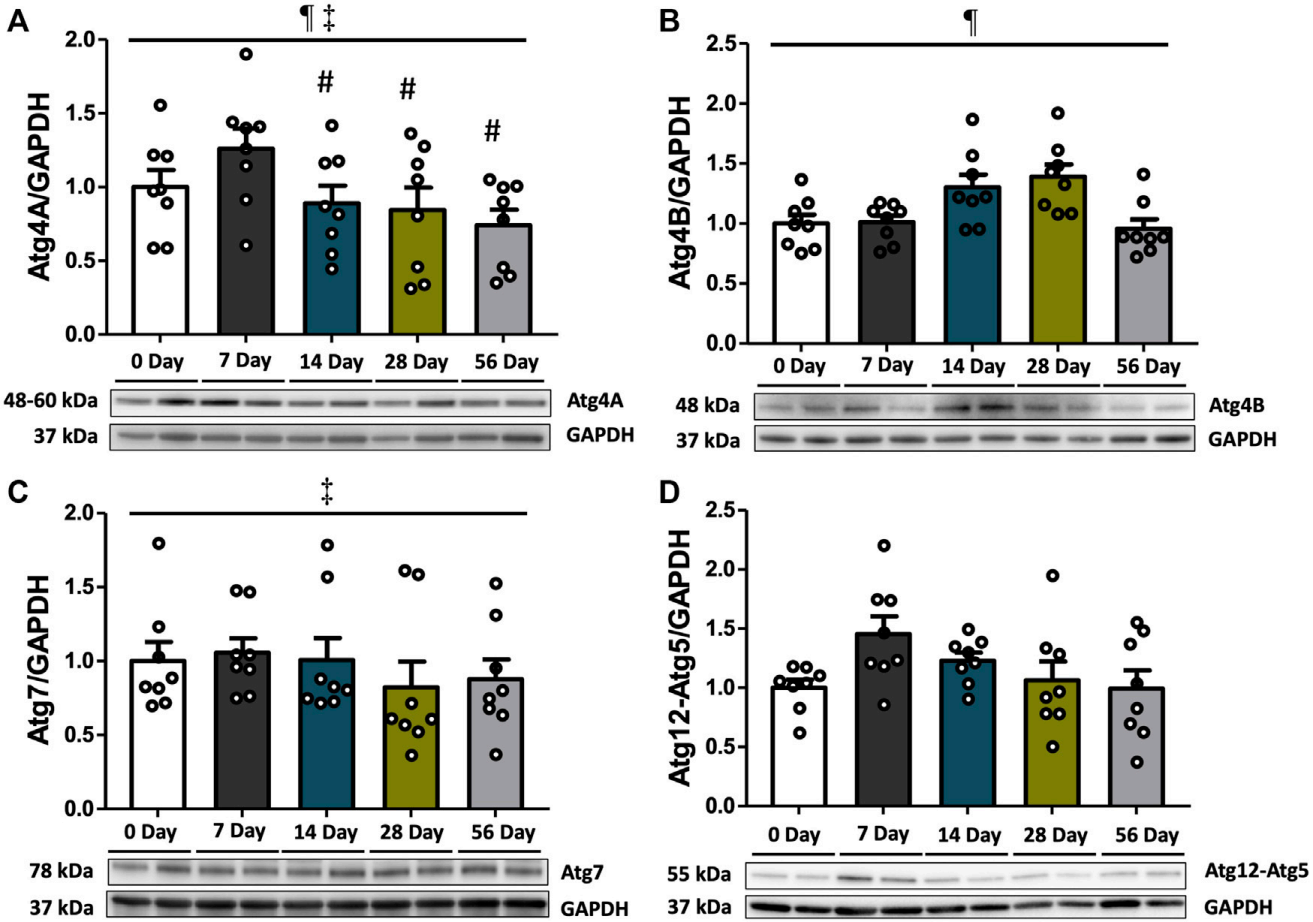
Pre-lysosomal markers subsequent to ACL injury. Protein concentrations of Atg4A, Atg4B, Atg7, and Atg12-Atg5 were measured by Western blot analysis with supportive representative images. **(A)** Atg4A protein contents were decreased at 14 days compared to 7 days, and this reduction persisted until 56 days. **(B)** Atg4B protein contents were increased at 14- and 28 days compared to controls and decreased at 56 days. **(C)** Atg7 protein contents did not show significant change during the recovery period. **(D)** Atg12-Atg5 protein contents were unchanged. Data are presented as Mean ± SEM. * Significant difference (*p* < 0.05) *versus* CON. # Significant difference (*p* < 0.05) *versus* 7 days. § Significant difference (*p* < 0.05) *versus* 14 days. † Significant difference (*p* < 0.05) *versus* 28 days. ¶ Significant main effect of time regardless of sex (*p* < 0.05). ‡ Significant main effect of sex regardless of time (*p* < 0.05).

To investigate the elongation of autophagosome following Beclin1-induced nucleation, we assessed the protein expression levels of Atg4A, Atg4B, Atg7, and Atg12-Atg5 (Figure 2). Atg4B was found to be increased by 1.3- and 1.4-fold at 14- and 28 days, respectively, compared to both controls and 7 days, and decreased afterward at 56 days relative to 14- and 28 days (*p* < 0.05, Figure 2B). Compared to the 7-day time point, Atg4A levels exhibited a 1.4-fold decrease at 14 days (*p* < 0.05), and this significant decline was remained up to 56 days post-injury (*p* < 0.05, Figure 2A). However, this finding should be taken into context as no post-injury differences were found between Atg4A and controls (*p* > 0.05). Irrespective of time and injury status, Atg4A levels were also higher in females than in males (*p* = 0.003, Supplementary Figure S2A). Atg7 levels were not affected by time or injury status (*p* > 0.05, Figure 2C), however, females demonstrated higher basal levels of Atg7 compared to males (*p* = 0.002, Supplementary Figure S2C). No differences were observed in Atg12-Atg5 levels (Figure 2D).

### 3.3 Autophagosome markers LC3 and p62 increased autophagy flux at 14 days following ACL injury

**FIGURE 3:**
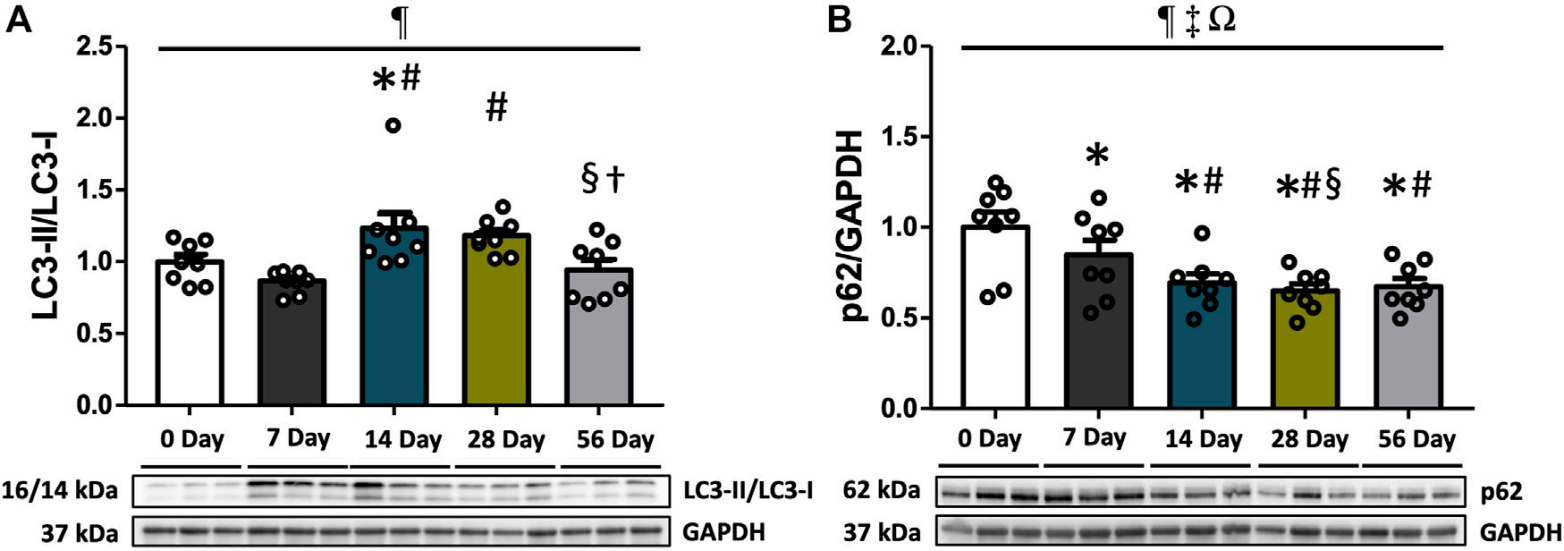
Autophagosome markers following ACL injury. LC3-II/LC3-I ratio and p62 protein concentrations were quantified using Western blot analysis with supportive representative images. **(A)** LC3-II/LC3-I ratio was significantly increased at 14- and 28 days compared to CON and 7 days, and decreased at 56 days. **(B)** p62 expressions were significantly reduced at 7 days and remained suppressed until 56 days. Data are presented as Mean ± SEM. * Significant difference (*p* < 0.05) *versus* CON. # Significant difference (*p* < 0.05) *versus* 7 days. § Significant difference (*p* < 0.05) *versus* 14 days. † Significant difference (*p* < 0.05) *versus* 28 days. ¶, ‡, Ω Significant main effects of time, sex, and their interaction (*p* < 0.05).

LC3-II, which is derived from the conversion of LC3-I, acts as a marker for autophagosomes. It directly interacts with p62, functioning as a cargo receptor for ubiquitinated substrates. To evaluate autophagy flux, we measured the protein expression levels of LC3-II, LC3-I, and p62. Additionally, the LC3-II/LC3-I ratio was calculated. The analysis revealed that the LC3-II/LC3-I ratio increased over time (*p* = 0.003), with significantly elevated levels at 14- and 28 days compared to controls and 7 days (*p* < 0.05, Figure 3A). Subsequently, the ratio decreased and returned to normal levels at 56 days compared to 14- and 28 days (*p* < 0.05). No significant difference was observed between sexes (Figure 3A). Regarding p62 levels, they exhibited variations based on time and sex (interaction *p* = 0.035, Figure 3B). Post hoc analyses indicated that females had significantly lower p62 levels at 7- and 14 days post-injury compared to males (*p* < 0.05, Supplementary Figure S3B). Moreover, p62 levels were consistently reduced at all time points following injury relative to controls and 7 days (*p* < 0.05, Figure 3B), with no significant difference in magnitude beyond 14 days (Figure 3B). Irrespective of time and injury status, males consistently exhibited higher p62 levels compared to females (*p* < 0.001, Supplementary Figure S3B).

### 3.4 PINK1-parkin-mediated mitophagy markers emerged after 7 days and maintained high levels during the later recovery period

**FIGURE 4:**
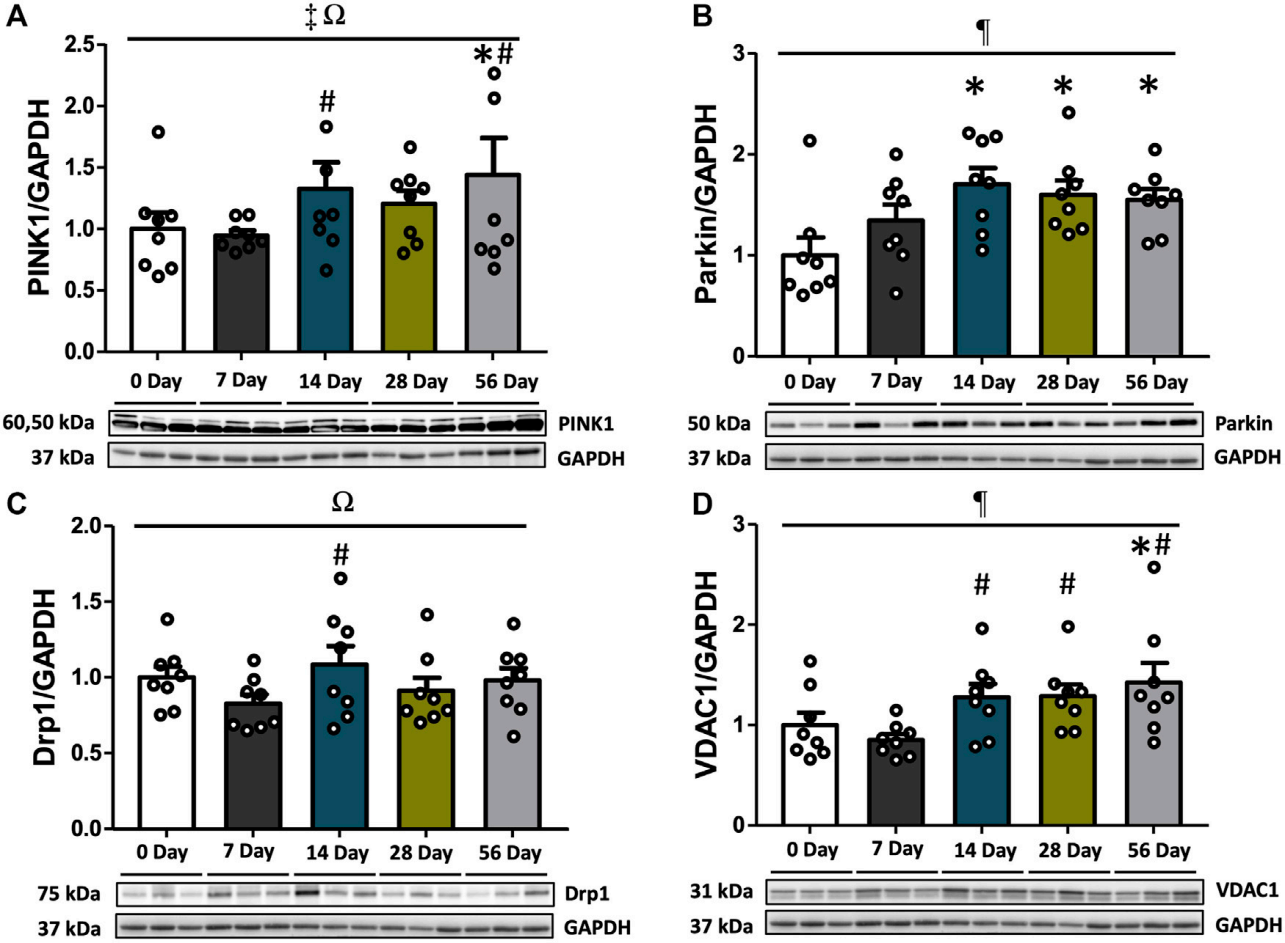
PINK1-Parkin-mediated mitophagy markers over the time course of ACL injury. PINK1, Parkin, Drp,1 and VDAC1 protein concentrations were measured by Western blot analysis with supportive representative images. **(A)** PINK1 levels were increased at 14 days relative to 7 days. PINK1 were upregulated at 56 days compared to CON and 7 days (*p* < 0.05). **(B)** Protein levels of Parkin were enhanced at 14 days compared to CON, which was maintained until 56 days. **(C)** Drp1 protein levels were increased at 14 days compared to 7 days. **(D)** VDAC1 protein levels were augmented at 14 days in comparison with 7 days and it was preserved until 56 days. VDAC1 levels at 56 days were significantly higher than CON. Data are presented as Mean ± SEM. * Significant difference (*p* < 0.05) *versus* CON. # Significant difference (*p* < 0.05) *versus* 7 days. ¶, ‡, Ω Significant main effects of time, sex, and their interaction (*p* < 0.05).

Mitophagy, which selectively eliminates damaged mitochondria through autophagosomes, involves the PINK1-Parkin pathway. We examined the protein expression of PINK1, Parkin, Drp1, and VDAC1 to assess alterations in mitophagy markers following ACL injury. PINK1 levels exhibited variations based on time and sex (interaction *p* = 0.013, Figure 4A). Post hoc analysis revealed significantly reduced levels of PINK1 in the female control and ACL-injured rats at 14- and 56 days compared to males (*p* < 0.05, Supplementary Figure S4A). Regardless of sex, PINK1 levels were upregulated by 1.4- and 1.5-fold at 56 days compared to controls and 7 days (*p* < 0.05, Figure 4A). Furthermore, males consistently exhibited higher PINK1 levels compared to females, irrespective of time and injury status (*p* < 0.001, Supplementary Figure S4A). Parkin displayed time-dependent increases (*p* = 0.029). Compared to controls, Parkin expression levels were elevated by 1.7-fold at 14 days, 1.6-fold at 28 days, and 1.5-fold at 56 days (*p* < 0.05), with no observed sex differences (Figure 4B). Drp1 levels varied based on time and sex (interaction *p* = 0.033). Post hoc analysis revealed significantly lower levels of Drp1 in males compared to females at 7 days post-injury (*p* = 0.001, Supplementary Figure S4C). Regardless of sex, the expression of Drp1 was upregulated by 1.3-fold at 14 days compared to 7 days (*p* < 0.05, Figure 4C). Notably, theses finding should be taken into context as no post-injury differences were found between Drp1 and controls (*p* > 0.05). Lastly, independent of sex, VDAC1 levels also increased by 1.4-fold at 56 days compared to controls (*p* < 0.05, Figure 4D). Significant increases in ACL-injured rat VDAC1 levels were observed after day 7 (*p* < 0.05, Figure 4D).

### 3.5 Lysosomal markers Lamp1 and Lamp2 were augmented after ACL injury

**FIGURE 5:**
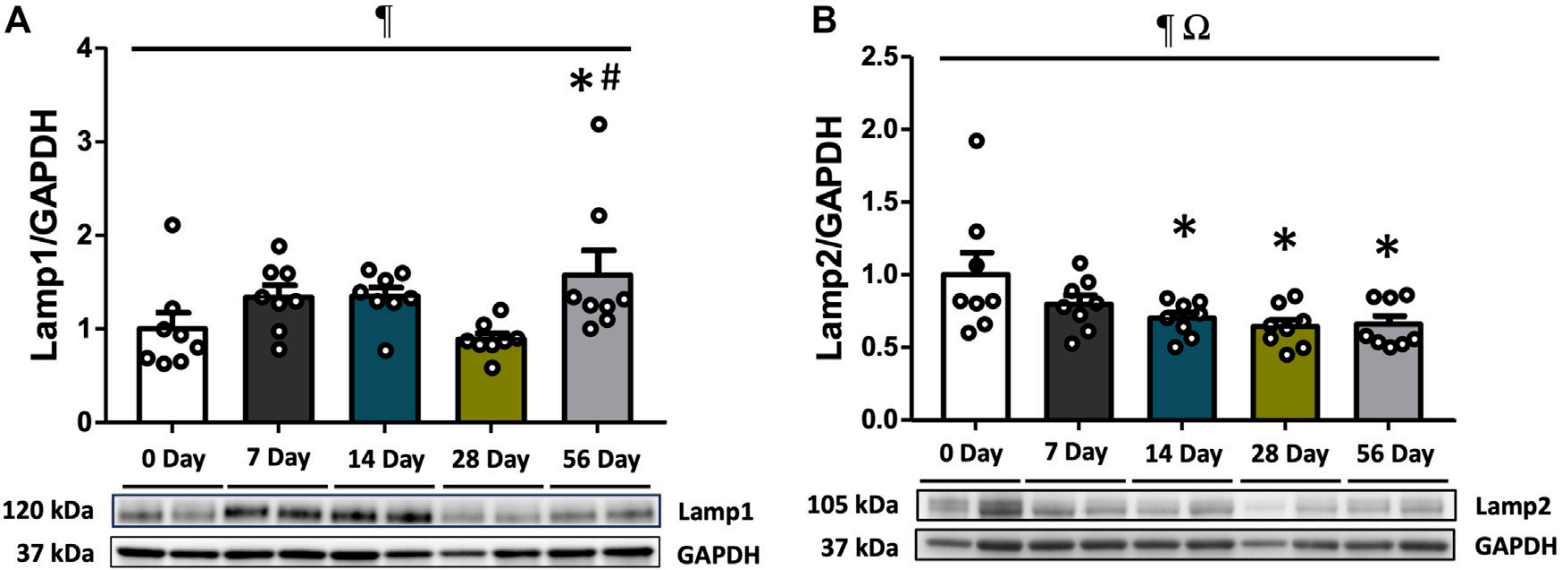
Lysosomal markers during recovery period after ACL injury. Lamp1 and Lamp2 protein concentrations were measured by Western blot analysis with supportive representative images. **(A)** Lamp1 protein contents were significantly higher at 56 days compared to CON and 28 days. **(B)** Lamp2 protein contents were diminished at 7 days in comparison with CON and it remained low until 56 days. Data are presented as Mean ± SEM. * Significant difference (*p* < 0.05) *versus* CON. † Significant difference (*p* < 0.05) *versus* 28 days. ¶ Significant main effect of time regardless of sex (*p* < 0.05). Ω Significant main effect of interaction between time and sex (*p* < 0.05).

As a crucial step in the maturation of autophagy processes, the fusion of lysosomes with autophagosomes results in the formation of autolysosomes, which are capable of degrading damaged organelles. To assess this stage of autophagy, the expressions of Lamp1 and Lamp2 were measured. Regardless of sex, Lamp1 levels were elevated by 1.8- and 2.0-fold at 56 days compared to controls and 28 days (*p* < 0.05, Figure 5A). Lamp2 levels were found to be dependent on both time and sex (interaction *p* = 0.011), and further analysis revealed significantly higher levels of Lamp2 in female rats at 56 days compared to males (*p* = 0.05, Supplementary Figure S5B). Following ACL injury, Lamp2 levels were decreased regardless of sex, with a 1.3-fold decline at 7 days compared to controls (*p* < 0.05), and this decrease persisted through 56 days (*p* < 0.05, Figure 5B).

## 4 Discussion

Earlier studies have highlighted the significance of mitochondrial homeostasis in muscle atrophy induced by immobilization, diseases, and trauma (Masiero et al., 2009; Romanello et al., 2010; Powers et al., 2012; Cannavino et al., 2015). However, the role of autophagy/mitophagy in the context of muscle weakness following ACL injury remains largely unexplored. In our previous study, we observed mitochondrial impairments in the quadriceps muscle after ACL rupture, characterized by increased ROS level and reduced oxygen consumption (Davi et al., 2022). In the same cohort of rats, we have also rigorously documented that there are severe declines in knee movement profiles after ACL injury that are directly linked with markers of knee joint health (White et al., 2022). Importantly, early clinical data also show disrupted mitochondrial respiratory capacity with prolonged oxidative damage after ACL injury in humans (Latham et al., 2024). Building upon these findings, the current study sought to elucidate the temporal progression of key quadricep muscle autophagy and mitophagy events after non-invasive ACL injury using non-invasive preclinical model. Our findings demonstrated dynamic changes in autophagy and mitophagy markers in the quadriceps muscle during the recovery period after ACL injury. The early response to the injury was characterized by the induction of autophagy at day 7, indicating an initial cellular response to the injury. Subsequently, at day 14, we observed fluctuations in the elongation of autophagosomes, suggesting a potential remodeling process. Notably, at day 56, markers associated with the elimination of damaged mitochondria were elevated, indicating possible ongoing cellular repair and restoration. Together, these data highlight the complexity of muscle recovery after ACL injury and the important overlooked role of autophagy and mitophagy in promoting the recovery process.

Autophagy, the cellular process of self-digestion, is triggered by various forms of cellular stresses (He and Klionsky, 2009; Kroemer et al., 2010). Within the class III phosphatidylinositol 3-kinase (PtdIns3K) complex, Beclin1 plays a crucial role in autophagosome formation and serves as a key modulator for initiating autophagy (He and Levine, 2010). Given the involvement of Beclin1 in the nucleation of phagophore, the initial stage of autophagy, we investigated changes in Beclin1 protein expression following ACL injury. Here we found a significant increase in Beclin1 protein expression at 7- and 14 days post-injury (Figure 1). This finding is consistent with prior research indicating elevated Beclin1 expression during period of modified physical activity. Specifically, a rise in Beclin1 expression has been observed at 14 days following hindlimb suspension (Zhang et al., 2018). Furthermore, other studies have demonstrated a concurrent decrease in Bcl-2, an antiapoptotic protein that inhibits Beclin1 activity (Levine et al., 2008), after the same duration of inactivity (Takahashi et al., 2017). Additionally, Beclin1 expression has been reported to increase following denervation (Triolo et al., 2022) or bed rest (Brocca et al., 2012), providing further evidence that signaling pathways promoting autophagy are active in the absence of regular physiological activity. Interestingly, our findings indicated consistently higher Beclin1 expression in male rats compared to females, suggesting possibly sex-specific variations in autophagy. Similar trends have been observed in studies utilizing ischemic stroke mouse model and human umbilical endothelial cells (Addis et al., 2014; Patrizz et al., 2021). In the context of autophagy initiation, these findings may imply that males potentially manifest a heightened susceptibility to events regulating autophagy activation. Altogether, these results suggest that a change or reduction in physical activity may trigger the activation of autophagy in vastus lateralis muscle after ACL injury.

The transition from nucleation to elongation represents a pivotal step in the process of autophagy. To investigate the elongation of autophagosomes following Beclin1-induced nucleation, we assessed the protein expression levels of key markers involved in the autophagy process, including Atg4A, Atg4B, Atg7, and Atg12-Atg5. In our study, we observed a significant increase in Atg4B expression levels at 14- and 28 days of post-injury (Figure 2B). This increase aligns with the findings of others that have reported greater mRNA expression of Atg4B in human vastus lateralis muscles after 14 days of immobilization (Suetta et al., 2012), and in atrophying mice 7 days after denervation (Zhao et al., 2007). The escalation of Atg4B expression suggests a potentially robust Atg4-mediated conversion of pre-LC3 to LC3-I, which is essential for autophagosome formation (Seibenhener et al., 2004) and conjugation of LC3 for LC3 recycling (Nakatogawa et al., 2012). Interestingly, while Atg4B was elevated, no difference between post-injury Atg4A expression and controls was found, though there was fluctuation in the expression between recovery days (Figure 2A). This discrepancy between Atg4A and Atg4B may stem from the differing affinity within the Atg4 family. Specifically, Atg4A exhibits a preference for GABARAP (i.e., a LC3 homolog), while Atg4B processes all LC3 homologs, serving as a key regulator of LC3/GABARAP conjugation system (Li et al., 2011). In accordance with our findings, others have shown that the re-expression of Atg4B sufficiently restores autophagic progression in cells lacking ATG4s, but sole Atg4A was insufficient for recovery (Nguyen et al., 2020). In the context of our work, this suggests that the high expression of Atg4B after ACL injury was likely sufficient for the development of Beclin1-mediated autophagy, independent of Atg4A. Other notable observations include the consistently higher levels of Atg4A in females compared to males, regardless of the time point or injury status. We also found that the expression levels of Atg7 were not significantly affected by time or injury status, though females had higher basal levels of Atg7 compared to males (Figure 2C; Supplementary Figure S2C). Further, we did not observe significant differences in the expression levels of Atg12-Atg5, another important complex involved in autophagosome elongation (Figure 2D). All together, these results suggest several plausible explanations for the observed maintenance or reduction in the expression levels of ATGs except for Atg4B, coupled with the upregulation of Beclin1 and mitophagy markers during recovery period. First, it is important to highlight that, among the two main conjugation systems, the LC3-PE conjugation system seems to be the dominant system in the quadriceps muscles post-ACL injury. This is supported by the heightened protein expressions of Atg4B and LC3 at 14 days, coupled with the comparatively steady levels of Atg7 and Atg12-Atg5 observed throughout the recovery period. Secondly, alterations in autophagic protein expression appear to be sex-specific, and in our study muted alterations in males could have been overshadowed by consistently high protein expression levels in females throughout the entire period. To this point, we observed that Atg4A and Atg7 exhibited sex-specific effects with higher expression levels in females than males. Furthermore, while Atg12-Atg5 expression in males was increased at 7 days compared to controls (Supplementary Figure S2D), changes in females remained relatively consistent. Collectively, our findings indicate that while the expressions of Atg4A, Atg7 and Atg12-Atg5 remain relatively stable in response to ACL injury, there is a noticeable increase in Atg4B expression, which may drive robust autophagy at 14- and 28 days post-injury.

To further explore the role of autophagy in ACL injury, we investigated the autophagy flux by examining the protein expression levels of LC3-II, LC3-I, and p62, along with calculating the LC3-II/LC3-I ratio. Previous studies have demonstrated an elevated LC3-II/LC3-I ratio following both hindlimb suspension (Takahashi et al., 2017; Zhang et al., 2018) and denervation (Memme et al., 2022). Consistent with these findings, our study revealed significantly increased LC3-II/LC3-I ratio at 14- and 28 days post-injury compared to controls and 7 days (Figure 3A). This suggests a higher conversion of LC3-I to LC3-II and a robust autophagosome formation at 14- and 28 days post-injury. Furthermore, we examined the expression of p62, also known as SQSTM1/sequestome1, which plays a role in delivering ubiquitinated cargoes to autophagosomes for degradation by autolysosomes (Bjorkoy et al., 2009; Liu et al., 2016). Notably, the level of p62 expression is known to be inversely correlated with autophagic activity, where increased p62 levels indicate autophagic suppression and decreased p62 levels indicate autophagic activation (Mizushima and Yoshimori, 2007). In our study, p62 was found to be consistently reduced at all time points following injury compared to controls and 7 days, indicating an active autophagic process (Figure 3B). Similar findings have also been observed in a rat hindlimb suspension study where p62 expression was reduced at 14 days (Zhang et al., 2018). Moreover, downregulation of p62 has been observed in other contexts of inactivity-induced autophagy, such as in cardiac muscle (Liu et al., 2015) and bone marrow stem cells (Zhou et al., 2018). When considering the changes in autophagosome markers, our data suggests that the autophagy flux, at least in terms of protein expression levels, was elevated between 14- and 28 days post ACL injury.

Mitophagy is a type of selective autophagy that specifically targets damaged or dysfunctional mitochondria (Ding and Yin, 2012). This process is facilitated by a decrease in membrane potential and an increase in the production of ROS (Okatsu et al., 2012; Triolo and Hood, 2021). Especially, elevated ROS levels can directly modify and activate Drp1, leading to mitochondrial fission and the formation of smaller mitochondrial fragments, some of which may contain damaged components (Shi et al., 2021). In response to damaged mitochondria and/or the loss of membrane potential, PINK1 accumulates on the outer mitochondrial membrane and selectively recruits Parkin, an E3 ligase (Narendra et al., 2010). This recruitment subsequently triggers the ubiquitination of VDAC1 (Geisler et al., 2010), which labels damaged mitochondria for their selective removal. VDAC1, a protein primarily located in the outer mitochondrial membrane is capable of forming pores in the outer membrane and interacts with Parkin in the presence of impaired mitochondria (Sun et al., 2012). Recognizing the significance of the PINK1-Parkin-mediated mitophagy pathway, we assessed the critical roles of PINK1, Parkin, Drp1, and VDAC1 following ACL injury. Notably, we observed distinct increases in the contents of PINK1, Parkin, Drp1, and VDAC1 at 14 days post-injury (Figures 4A–D). Furthermore, we found that the levels of PINK1, Parkin, and VDAC1 remained elevated from 14- to 56 days, which indicates an upregulation of PINK1-Parkin-mediated mitophagy during the later stages of recovery in the quadriceps muscle. Prolonged physical inactivity is known to induce mitophagy, contributing to the disruption of mitochondrial function and resulting in critical adverse effects in skeletal muscle (Leermakers et al., 2019). Our findings align with this concept, as we observed enhanced mitophagy markers following ACL injury. These results are also consistent with our previous observations of reductions in mitochondrial RCR accompanied by sharp rises in ROS production that occurred as early as of 7 days post ACL injury, and was sustained up to day 56 (Davi et al., 2022). Prior work in hindlimb suspension has also reported an upregulated PINK1, Parkin, and Drp1 protein expression at 14 days (Zhang et al., 2018). Similarly, elevated PINK1 levels have been observed at 14 days post-nerve injury (Shen et al., 2020), and a rise in Parkin after immobilization-induced physical inactivity at 14- and 28 days (Zhang et al., 2023). Anoxia/reoxygenation-induced cardiomyocyte autophagy has been shown to promote the expression of PINK1, Parkin, and VDAC1 (Yang et al., 2021). On the other hand, VDAC1 knockdown inhibited the recruitment of Parkin to defective mitochondria, leading to decreases in PINK1, Parkin, and VDAC1 levels (Yang et al., 2021). In agreement with our overall findings, mitophagy markers were observed to be elevated from 14 days and continued through 56 days. Therefore, this sustained increase in mitophagy may result in contributing to skeletal muscle atrophy.

In the context of autophagy, the role of lysosomal membrane proteins, including Lamp1 and Lamp2, becomes significant as they facilitate the fusion of autophagosomes containing damaged organelles with lysosomes, leading to the formation of autolysosomes (Huynh et al., 2007). Through this fusion process, Lamp1 and Lamp2 contribute to the selective degradation within the autolysosomes. Monitoring the levels of Lamp proteins can therefore provide valuable insight into the degree of autophagosome-lysosome fusion and, by extension, the progression of autophagy. In our study, we observed that Lamp1 expression was upregulated at 56 days post-injury (Figure 5A), which is in-line with our PINK1-Parkin-mediated mitophagy events. This suggests that the clearance of cellular waste, such as protein aggregates or damaged organelles, may be actively occurring at this time point. Reversely, Lamp2 expression was decreased at 14 days and remained unchanged until 56 days (Figure 5B). Notably, other work has also reported an increase in Lamp1 and a decrease in Lamp2 with ischemic injury, and further shown Lamp2 overexpression alleviated lysosomal destabilization (Cui et al., 2020). It indicates that the decline in Lamp2 protein expression in autophagic circumstances displays the impaired autophagosome degradation capacity. The underlying mechanism for the opposite alterations in Lamp1 and Lamp2 expression is unclear. However, these data suggest that the autophagy processes in this unique ACL injury model may exhibit different affinities to Lamp1 and Lamp2. Overall, our observations highlight the complex dynamics of lysosomal membrane protein expression in relation to autophagy, emphasizing the need for further investigations to elucidate the underlying mechanisms and their implications in the context of ACL injury.

One limitation of our study is that we assessed autophagy changes during ACL injury using static markers. Autophagy is a dynamic process, so the upregulation of autophagy-related proteins can stem from either increased autophagy flux or downstream blockage. To address this limitation, we simultaneously measured p62 along with the LC3-II/LC3-I ratio. However, further analysis is recommended for future studies to validate our findings regarding the elevation of mitophagy markers after 14 days of ACL injury. For example, employing colchicine treatment may provide a clearer indication of autophagy and mitophagy flux, in addition to assessing p62 and the LC3-II/LC3-I ratio. Additionally, measuring lysosomal hydrolase activity with cathepsins could offer a better understanding of lysosomal status. Incorporating live cell imaging with mitochondrial fission would also provide enhanced insight. While these assessments were not conducted in the current study, we have, for the first time, offered new insights into the temporal trends of autophagy and mitophagy markers over time following ACL injury.

## 5 Conclusion

Muscle atrophy following ACL injury is distinct and accompanied with loss of muscle mass and function. We demonstrate, for the first time, the longitudinal changes in autophagy and mitophagy markers after ACL injury. The current study contributes to our understanding by providing evidence that autophagy and mitophagy markers were increased in quadriceps muscle after 14 days of non-invasive ACL injury. Moreover, the observed upregulation mitophagy markers coincided with impaired mitochondrial respiratory function and elevated levels of ROS, as previously reported. These findings may have potential implications for the development of mitochondria-targeted therapies to mitigate muscle atrophy following ACL injury.

## Data Availability

The original contributions presented in the study are included in the article/Supplementary Material, further inquiries can be directed to the corresponding authors.

## Supplementary material

SUPPLEMENTARY FIGURE S1 The upstream marker of autophagy following ACL injury. Beclin1 protein concentrations via western blot analysis with supportive representative images. Beclin1 levels exhibited an increase at 7- and 14 days compared to CON, followed by a decrease at 28- and 56 days. Data are presented as Mean ± SEM. * Significant difference (*p* < 0.05) versus CON. # Significant difference (*p* < 0.05) versus 14 days. ¶ Significant main effect of time regardless of sex (*p* < 0.05). ‡ Significant main effect of sex regardless of time (*p* < 0.05).

SUPPLEMENTARY FIGURE S2 Pre-lysosomal markers subsequent to ACL injury. Protein concentrations of Atg4A, Atg4B, Atg7, and Atg12-Atg5 were measured by western blot analysis with supportive representative images. **(A)** Atg4A protein contents were decreased at 14 days compared to 7 days, and this reduction persisted until 56 days. **(B)** Atg4B protein contents were increased at 14- and 28 days compared to controls and decreased at 56 days. **(C)** Atg7 protein contents did not show significant change during the recovery period. **(D)** Atg12-Atg5 protein contents were unchanged. Data are presented as Mean ± SEM. * Significant difference (*p* < 0.05) versus CON. # Significant difference (*p* < 0.05) versus 7 days. § Significant difference (*p* < 0.05) versus 14 days. † Significant difference (*p* < 0.05) versus 28 days. ¶ Significant main effect of time regardless of sex (*p* < 0.05). ‡ Significant main effect of sex regardless of time (*p* < 0.05).

SUPPLEMENTARY FIGURE S3 Autophagosome markers following ACL injury. LC3-II/LC3-I ratio and p62 protein concentrations were quantified using western blot analysis with supportive representative images. **(A)** LC3-II/LC3-I ratio was significantly increased at 14- and 28 days compared to CON and 7 days, and decreased at 56 days. **(B)** p62 expressions were significantly reduced at 7 days and remained suppressed until 56 days. Data are presented as Mean ± SEM. * Significant difference (*p* < 0.05) versus CON. # Significant difference (*p* < 0.05) versus 7 days. § Significant difference (*p* < 0.05) versus 14 days. † Significant difference (*p* < 0.05) versus 28 days. ¶, ‡, Ω Significant main effects of time, sex, and their interaction (*p* < 0.05). α Significant sex difference (*p* < 0.05) after injury (i.e., sex by time interaction).

SUPPLEMENTARY FIGURE S4 PINK1-Parkin-mediated mitophagy markers over the time course of ACL injury. PINK1, Parkin, Drp,1 and VDAC1 protein concentrations were measured by western blot analysis with supportive representative images. **(A)** PINK1 levels were increased at 14 days relative to 7 days. PINK1 were upregulated at 56 days compared to CON and 7 days (*p* < 0.05). **(B)** Protein levels of Parkin were enhanced at 14 days compared to CON, which was maintained until 56 days. **(C)** Drp1 protein levels were increased at 14 days compared to 7 days. **(D)** VDAC1 protein levels were augmented at 14 days in comparison with 7 days and it was preserved until 56 days. VDAC1 levels at 56 days were significantly higher than CON. Data are presented as Mean ± SEM. * Significant difference (*p* < 0.05) versus CON. # Significant difference (*p* < 0.05) versus 7 days. ¶, ‡, Ω Significant main effects of time, sex, and their interaction (*p* < 0.05). α Significant sex difference (*p* < 0.05) after injury (i.e., sex by time interaction).

SUPPLEMENTARY FIGURE S5 Lysosomal markers during recovery period after ACL injury. Lamp1 and Lamp2 protein concentrations were measured by western blot analysis with supportive representative images. **(A)** Lamp1 protein contents were significantly higher at 56 days compared to CON and 28 days. **(B)** Lamp2 protein contents were diminished at 7 days in comparison with CON and it remained low until 56 days. Data are presented as Mean ± SEM. * Significant difference (*p* < 0.05) versus CON. † Significant difference (*p* < 47 0.05) versus 28 days. ¶ Significant main effect of time regardless of sex (*p* < 0.05). Ω Significant main effect of interaction between time and sex (*p* < 0.05). α Significant sex difference (*p* < 0.05) after injury (i.e., sex by time interaction).
