# Supplementary figures and images for: Quadriceps muscle atrophy after non-invasive anterior cruciate ligament injury: evidence linking to autophagy and mitophagy
Source: Front Physiol. 2024 Mar 1;15:1341723. doi: 10.3389/fphys.2024.1341723 (PMC10940348; doi:10.3389/fphys.2024.1341723)

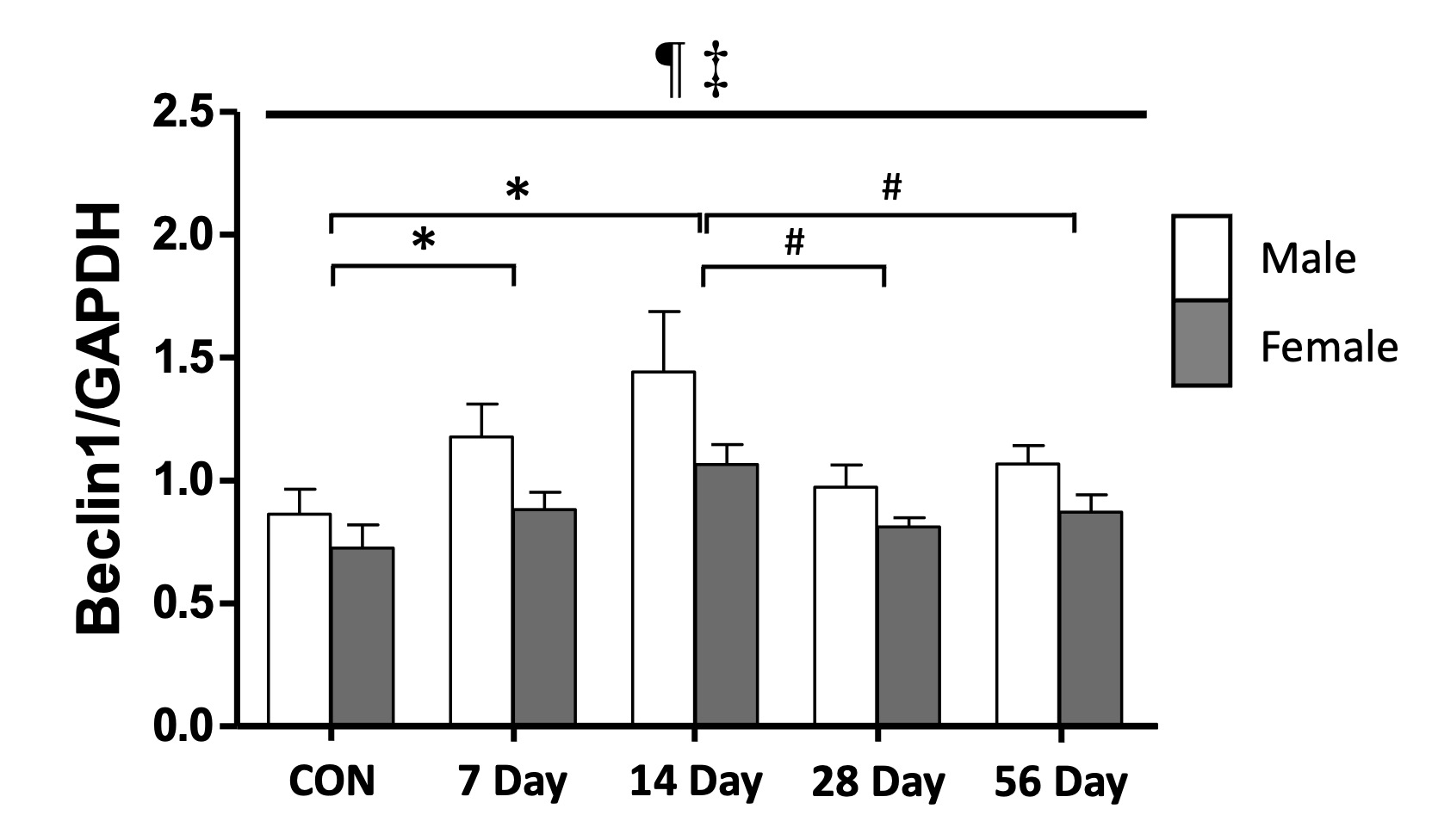

Supplement: Supplementary file 1 [file Presentation1.zip › Supplementary Material Presentation/Supplementary Figurec S1.jpg]

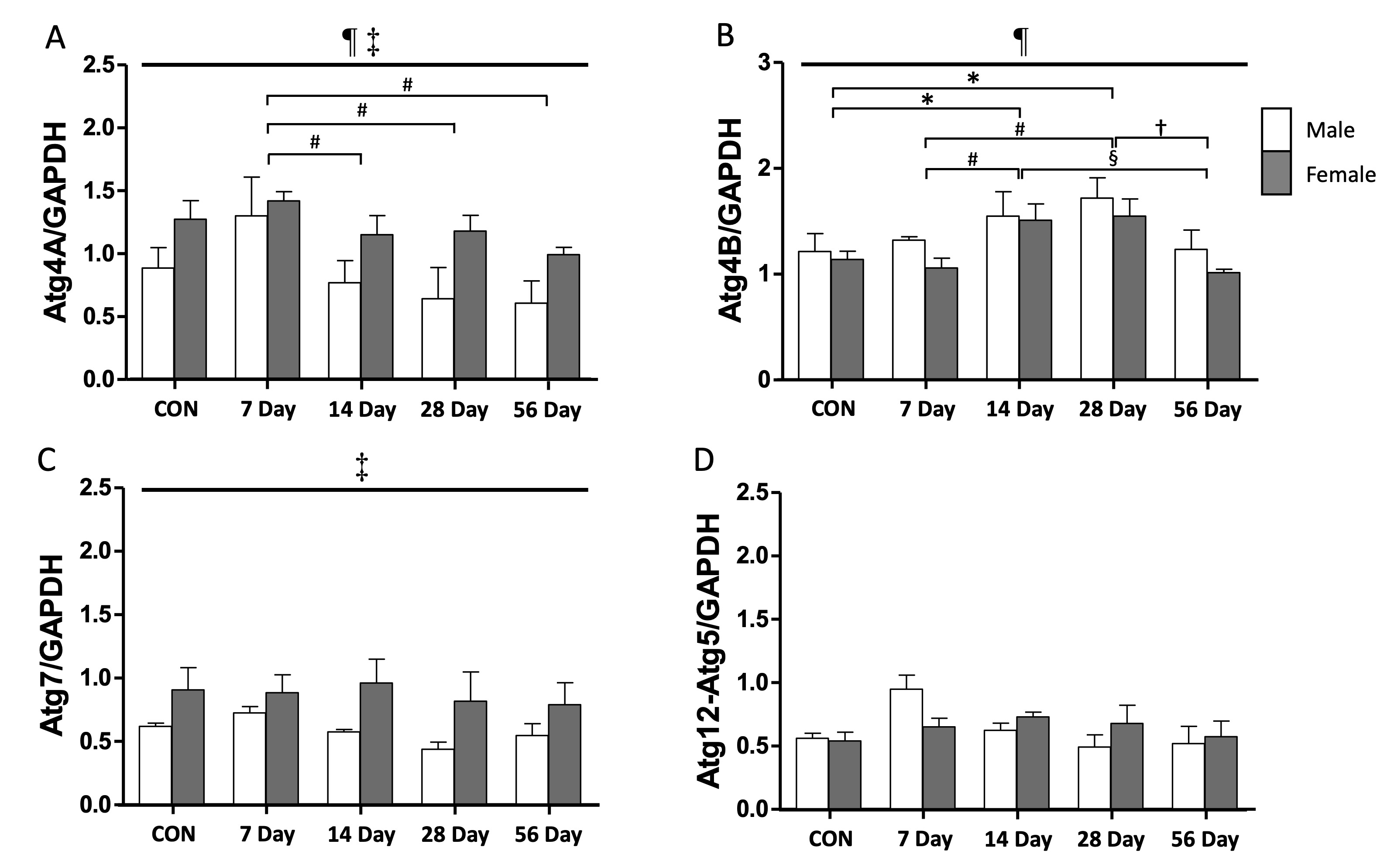

Supplement: Supplementary file 1 [file Presentation1.zip › Supplementary Material Presentation/Supplementary Figurec S2.jpg]

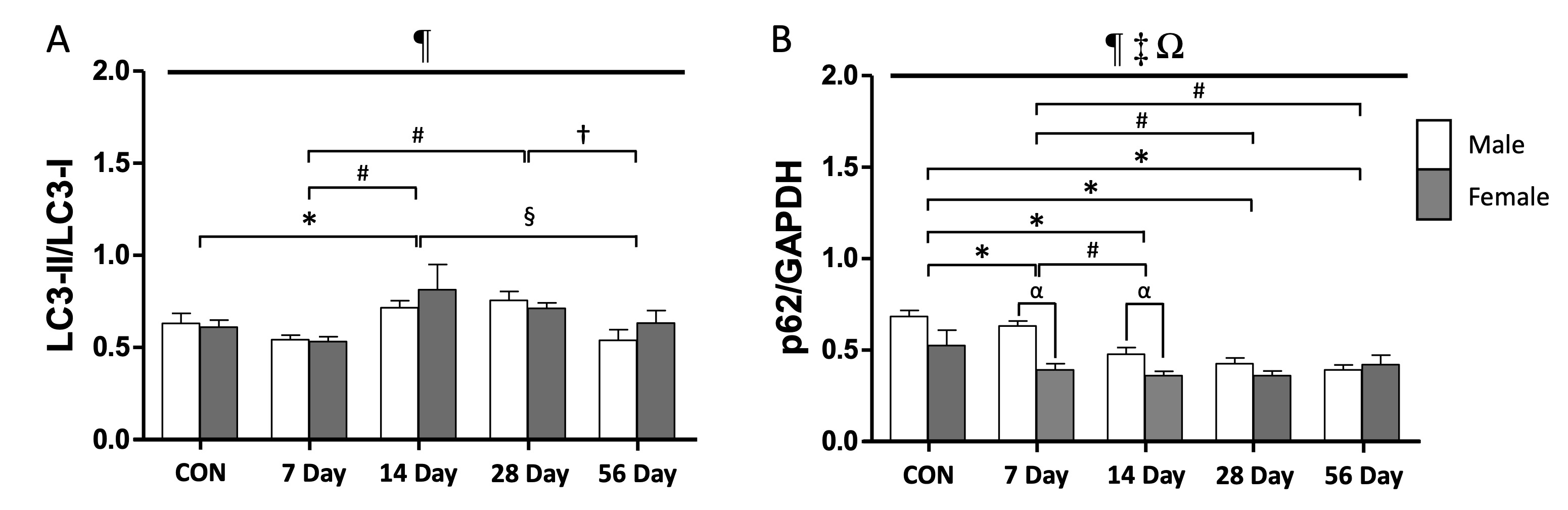

Supplement: Supplementary file 1 [file Presentation1.zip › Supplementary Material Presentation/Supplementary Figurec S3.jpg]

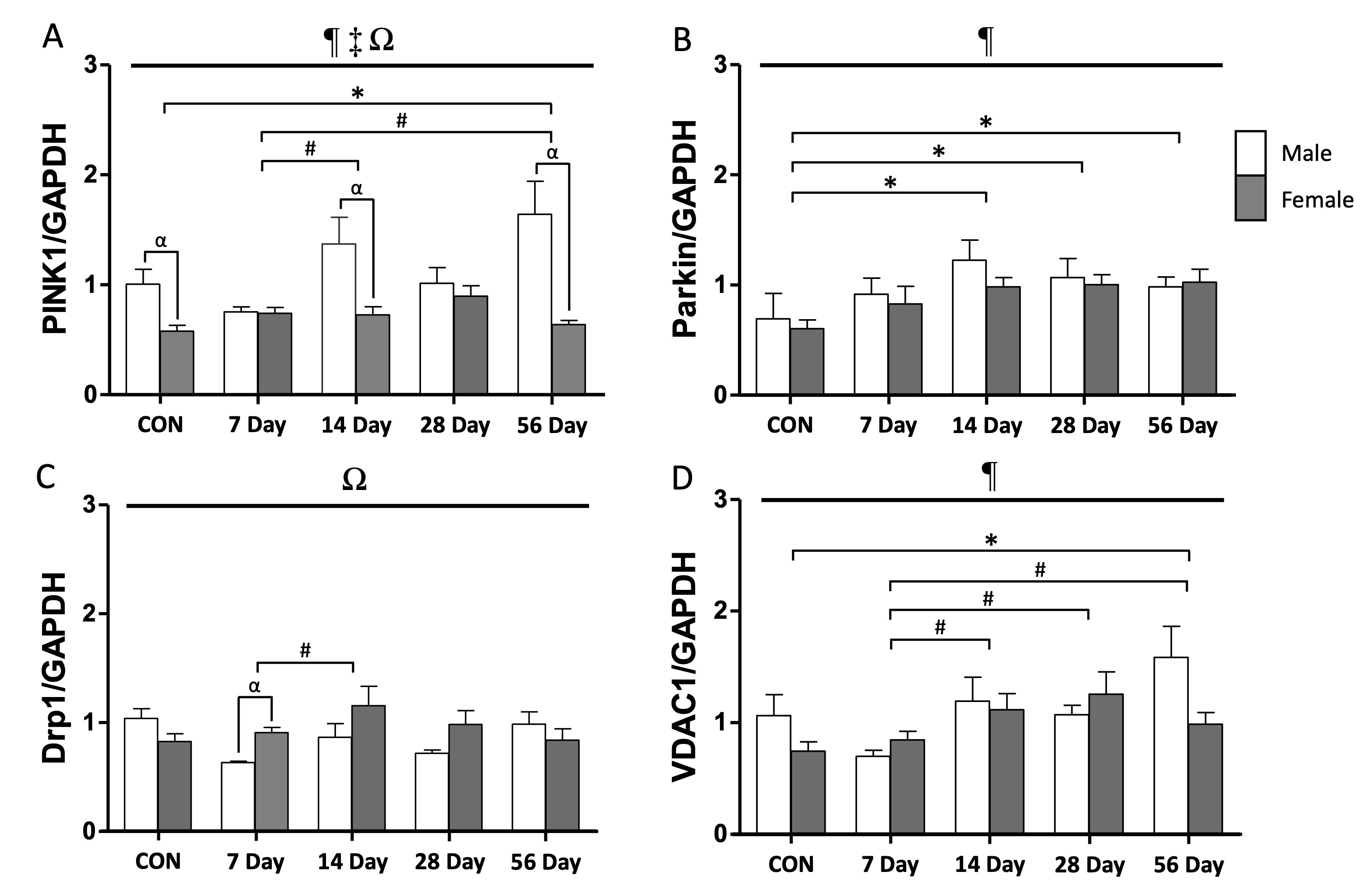

Supplement: Supplementary file 1 [file Presentation1.zip › Supplementary Material Presentation/Supplementary Figurec S4.jpg]

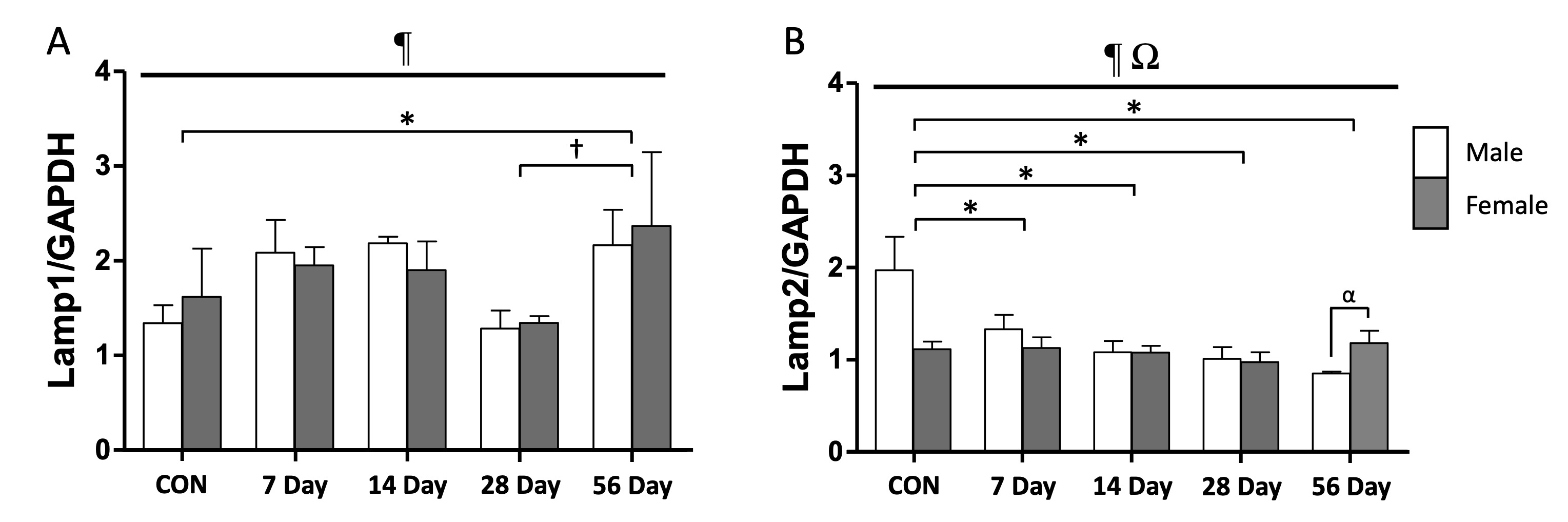

Supplement: Supplementary file 1 [file Presentation1.zip › Supplementary Material Presentation/Supplementary Figurec S5.jpg]
